# Supplementary material for: Development of pathophysiologically relevant models of sickle cell disease and β-thalassemia for therapeutic studies
Source: Nat Commun. 2024 Feb 27;15:1794. doi: 10.1038/s41467-024-46036-x (PMC10899644; doi:10.1038/s41467-024-46036-x)
Supplement: Supplementary file 1 — Supplementary Information [file 41467_2024_46036_MOESM1_ESM.pdf]

Development of pathophysiologically relevant models of sickle cell disease and β-thalassemia for therapeutic studies

Supplementary Fig.1

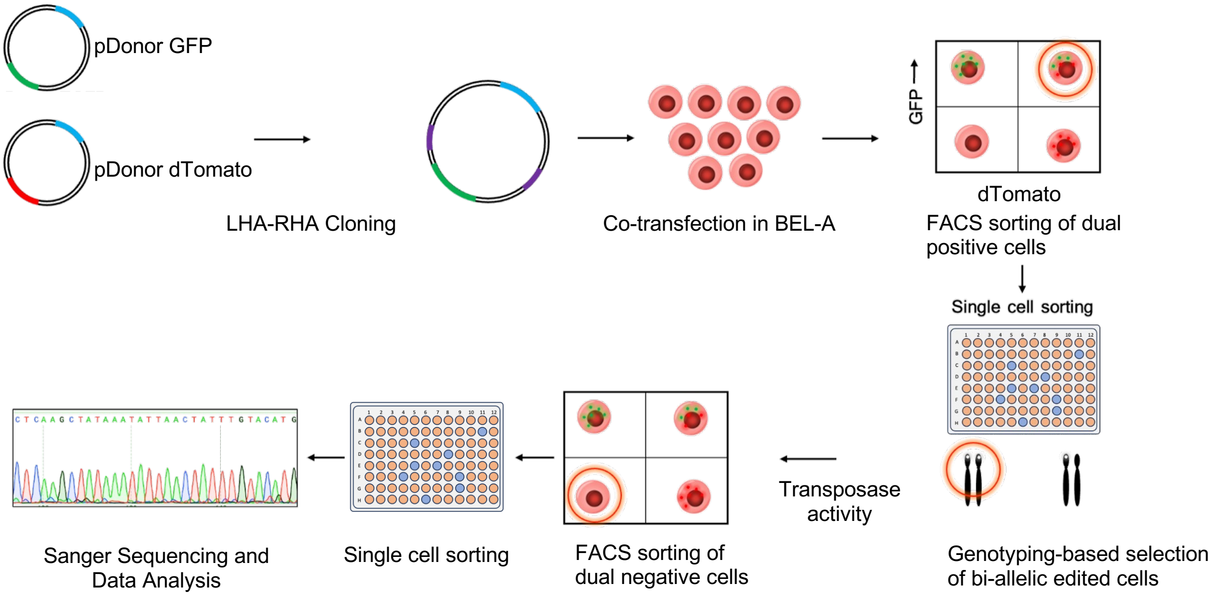

Supplementary Fig.1: Schematics demonstrating the generation of BEL-A SCM and BEL-A BTM disease cell lines using CRISPR-Cas9 coupled PiggyBac transposon system. BEL-A cells were transfected with pDONOR-eGFP-SCM/pDONOR-eGFP-BTM and pDONOR-dTomato-SCM/pDONOR-dTomato-BTM with sgRNA targeting HBB gene. eGFP+dTomato+ cells were bulk sorted followed by single cell sorting. Cells were screened using flow cytometry followed by PCR and Sanger confirmation. Confirmed clones were treated with transposase plasmid. eGFP-dTomato- cells were then bulk sorted followed by single cell sorting. Clones were screened using flow cytometry, PCR, enzyme digestion and confirmed through Sanger sequencing.

Supplementary Fig.2

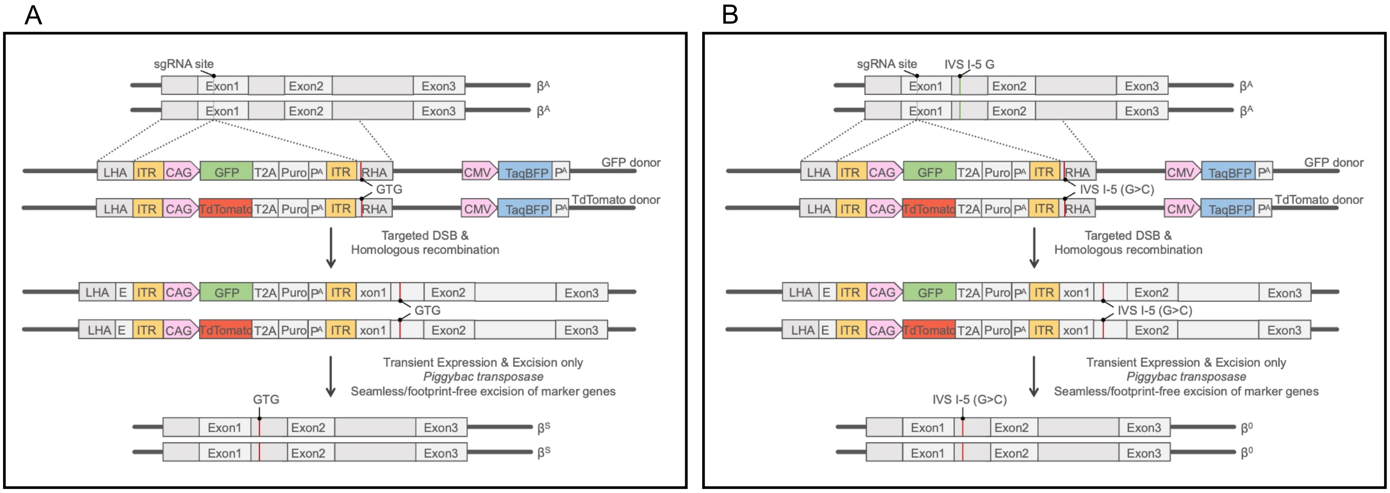

Supplementary Fig.2 : Schematics demonstrating HBB gene and the generation of **(A)** BEL-A SCM and **(B)** BEL-A BTM line.

Supplementary Fig.3

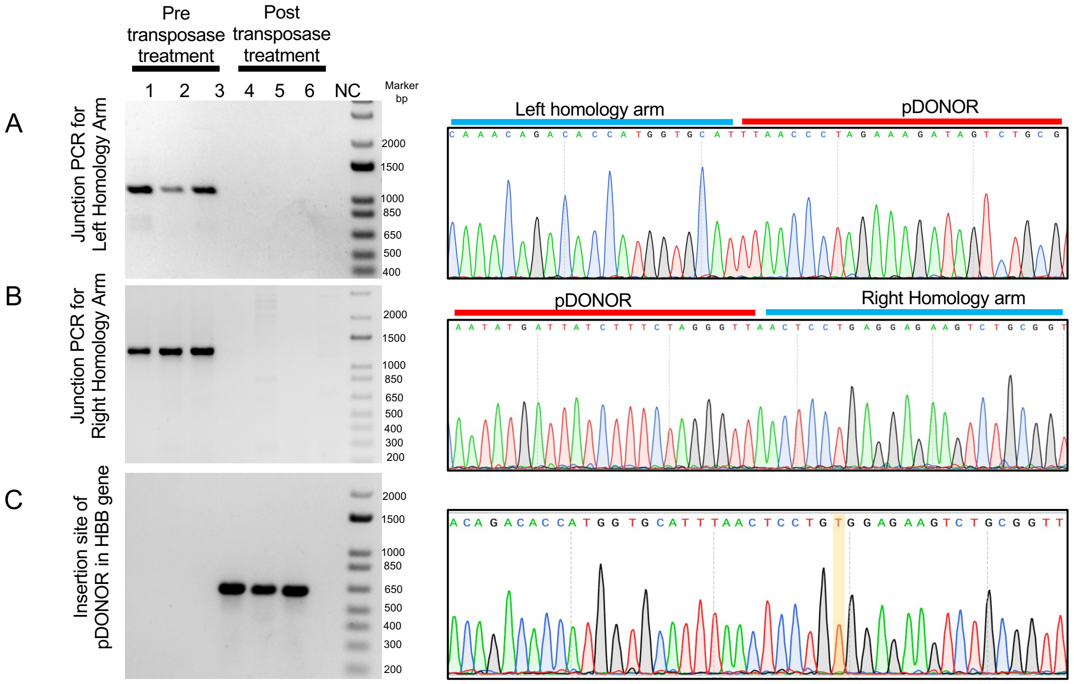

Supplementary Fig.3: Confirmation of BEL-A SCM clones at pre and post transposase treatment. Left and Right homology arm junction PCR and insertion site PCR **(A, B, C, Left)** suggest insertion at the target locus. Chromatogram **(A, B, right)** obtained from Sanger sequencing confirms the insertion at target locus. No amplification of Left and Right homology arm junction PCR post transposase treatment and amplification using insertion site primer suggest removal of PiggyBac cassette from the locus of interest. Sanger Sequencing chromatogram confirms footprint-free incorporation of sickle cell mutation (highlighted in yellow) in BEL-A cells (C,right).

Supplementary Fig.4

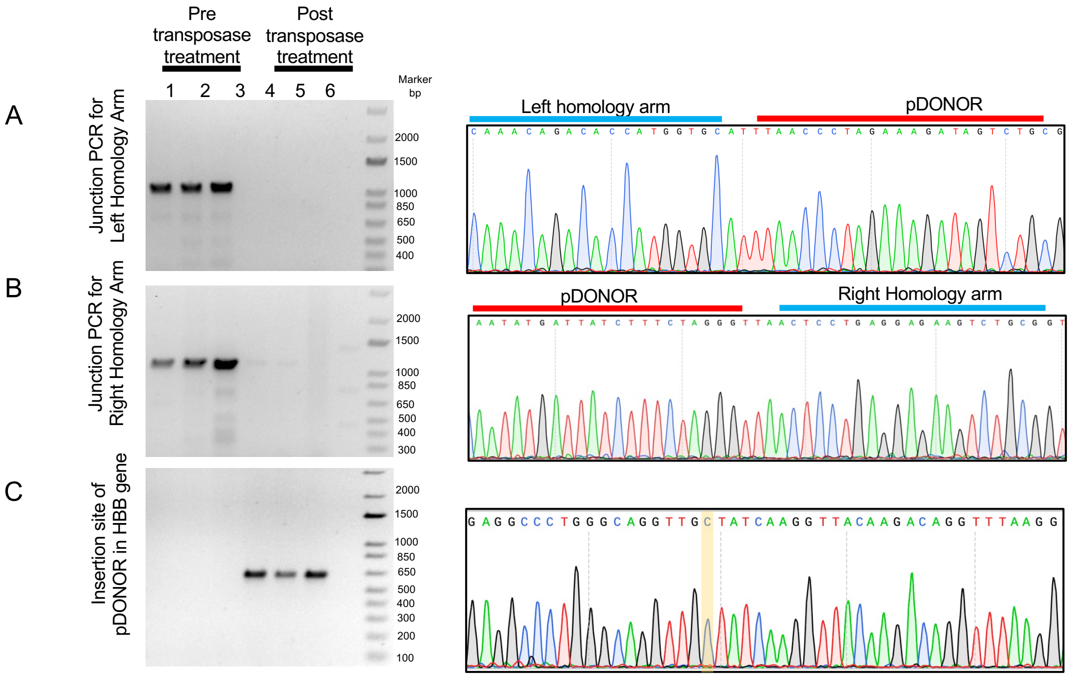

Supplementary Fig.4: Confirmation of BEL-A BTM clones at pre and post transposase treatment. Left and Right homology arm junction PCR and insertion site PCR **(A, B, C, Left)** suggest insertion at the target locus. Chromatogram **(A, B, right)** obtained from Sanger sequencing confirms the insertion at target locus. No amplification of Left and Right homology arm junction PCR post transposase treatment and amplification using insertion site primer suggest removal of PiggyBac cassette from the locus of interest. Sanger sequencing chromatogram confirms footprint-free incorporation of β- thalassemia IVS 1-5 mutation (highlighted in yellow) in BEL-A cells (C, right).

Supplementary Fig.5

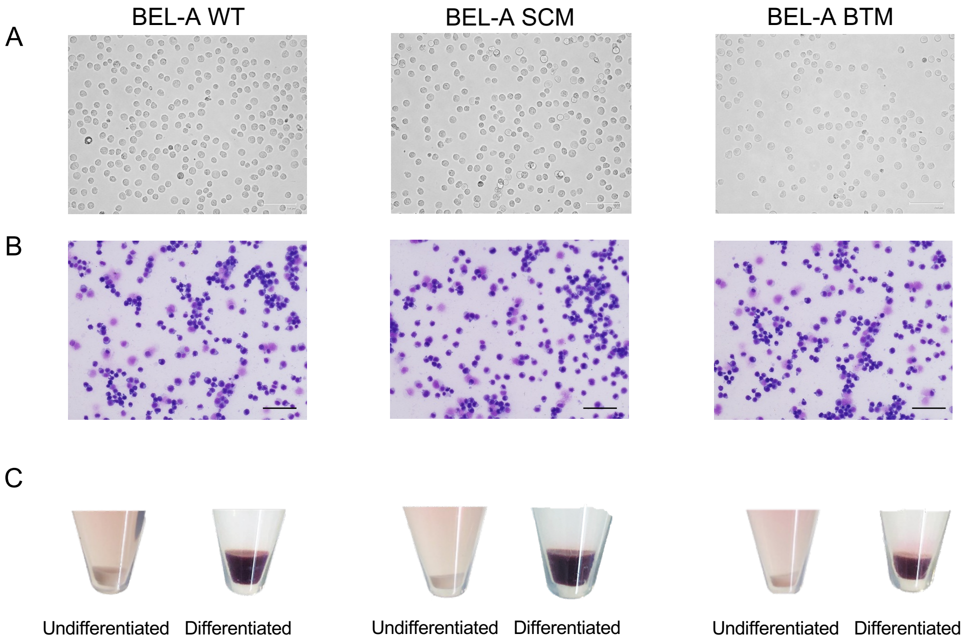

Supplementary Fig.5: Morphological analysis of BEL-A SCM and BEL-A BTM as compared to BEL-A WT cells. **(A)** Brightfield microscopic images of BEL-A WT, BEL-A SCM and BEL-BTM. Scale:100 µm. **(B)** Representative Giemsa images of BEL-A WT, BEL-A SCM and BEL-A BTM showing all three cells are at proerythroblast stage; Scale: 100 µm. **(C)** Pellet color of undifferentiated and differentiated BEL-A WT, BEL-A SCM and BEL-A BTM cells demonstrating differentiation potential of the disease model cell lines.

Supplementary Fig.6

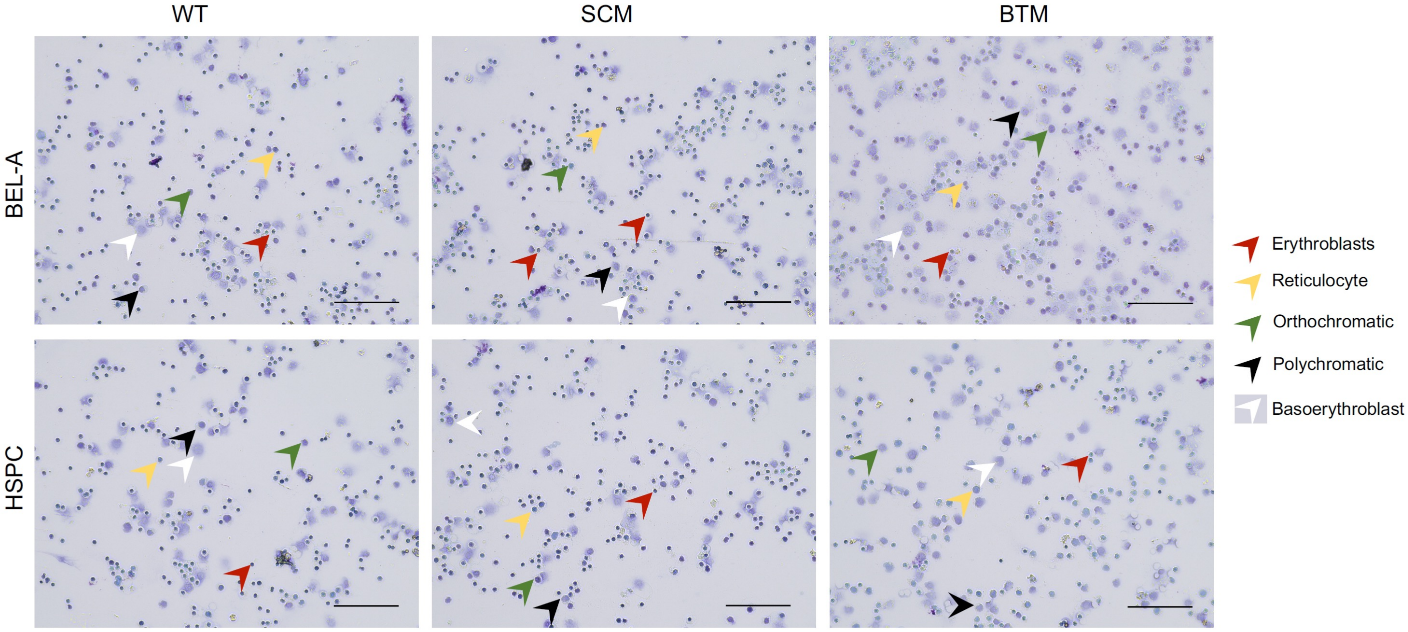

Supplementary Fig.6: Representative Giemsa stained images of BEL-A **(WT, SCM and BTM)** at Day 10 of differentiation and HSPCs **(WT, SCM and BTM)** at day 19 of differentiation. Experiment was done in three independent replicates, n=3.. White arrows indicate basoerythroblast, Black arrows indicate polychromatic erythroblast, Green arrows indicate orthochromatic erythroblast, Yellow arrows indicate reticulocyte and Red arrow indicate erythrocyte. Scale: 100 µm

Supplementary Fig.7

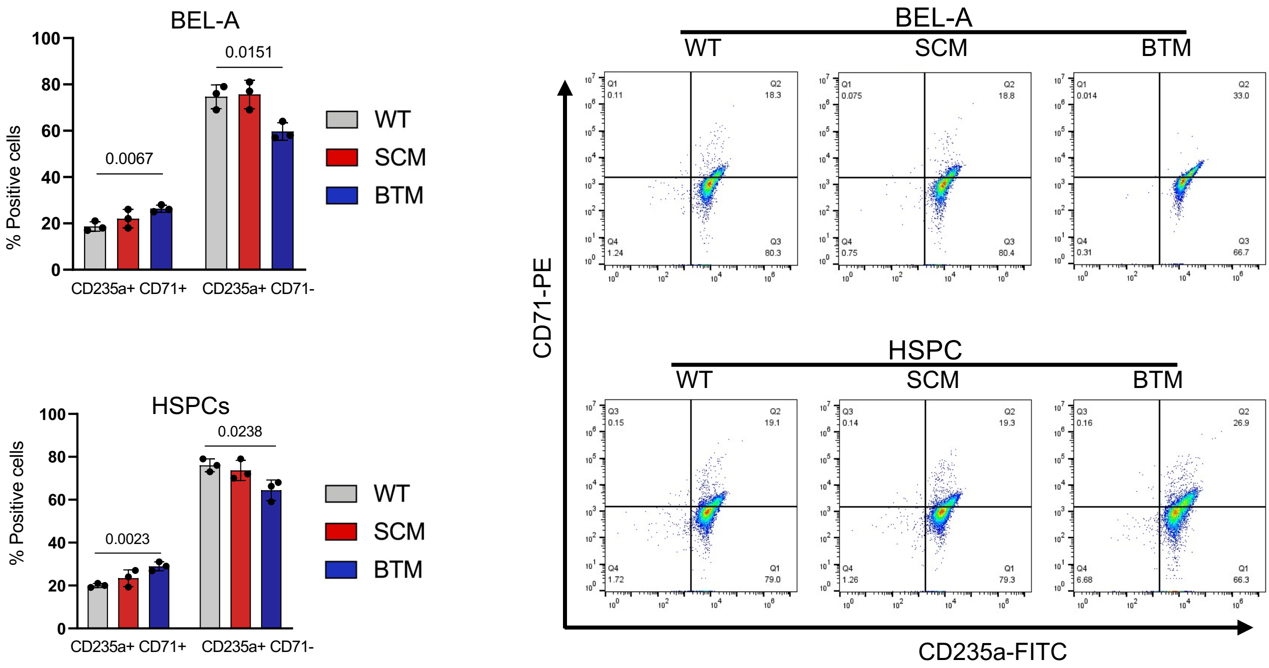

Supplementary Fig.7: Flow cytometric analysis to determine the percentage of erythroid differentiation markers, CD235a and CD71 in BEL-A (WT, SCM and BTM) and HSPCs (WT, SCM and BTM). Experiments were done in three independent replicates, n=3 and statistical significance was determined by two-tailed Student's t-test. Source data is provided in source file.

Supplementary Fig.8

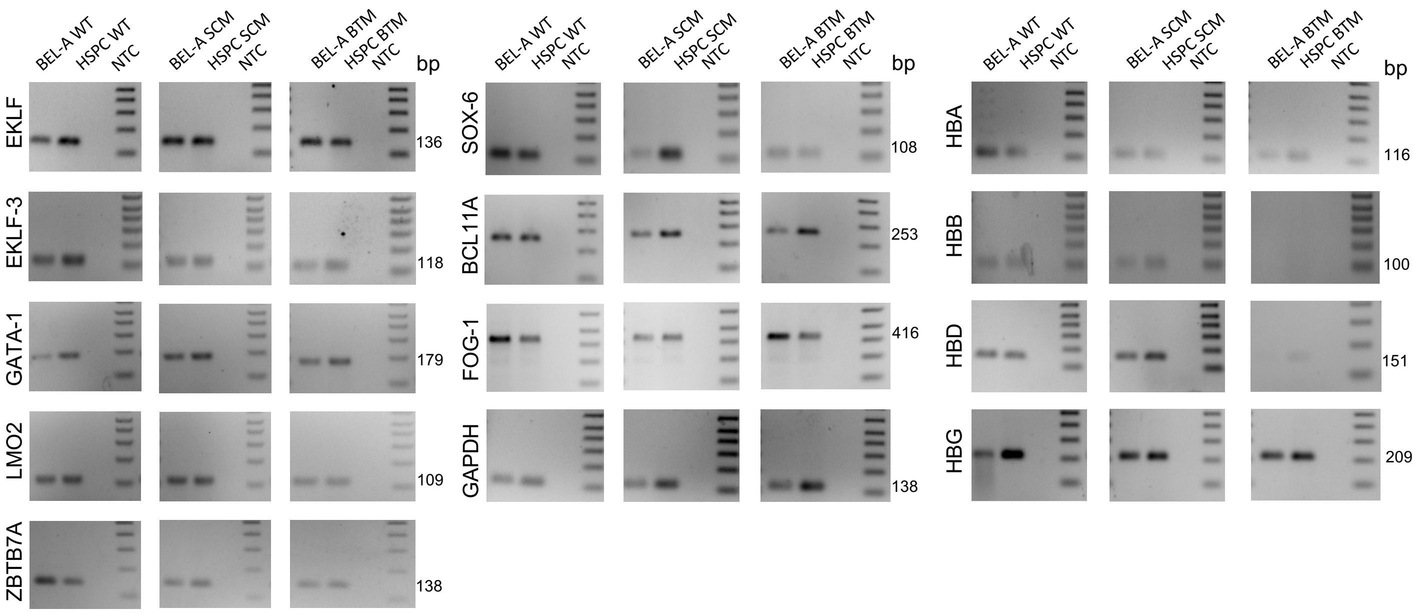

Supplementary Fig.8: Representative gel images of Semi-quantitative RT-PCR for key transcription factors and genes involved in erythropoiesis. Expression of the genes was analyzed at Day 6 of differentiation . The amplicon size of each gene is mentioned on right (in bp) and Supplementary Table 7. The experiment was performed three time independently, n=3.

Supplementary Fig.9

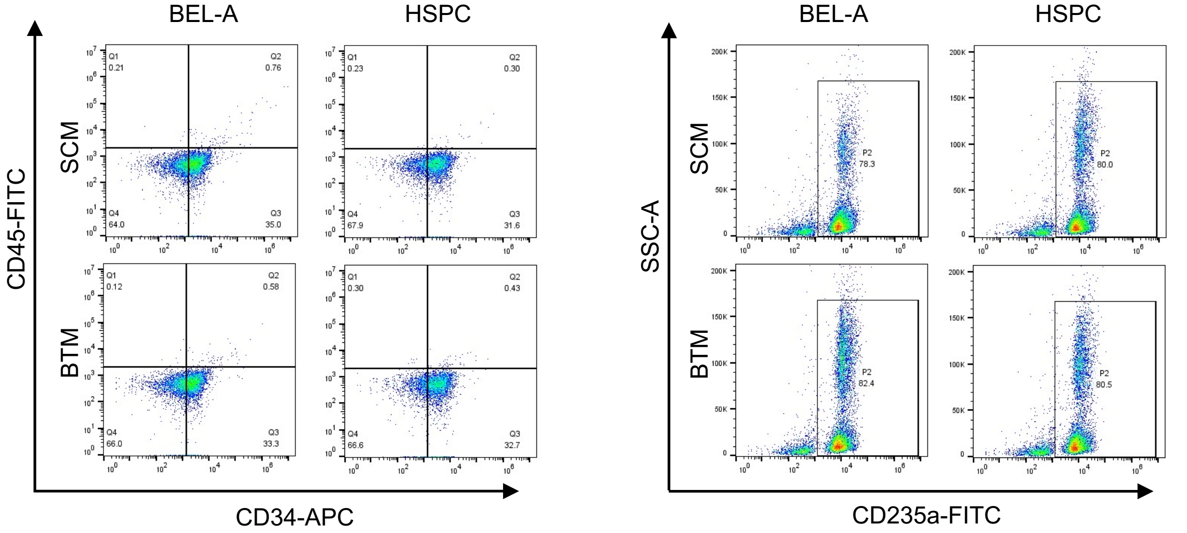

Supplementary Fig.9: Stage-matching of HSPCs-derived erythroid progenitor cells and BEL-A cells using erythroid lineage markers: CD45, CD34 (Left) and CD235a (Right). The experiment was performed three times independently, n=3.

Supplementary Fig.10

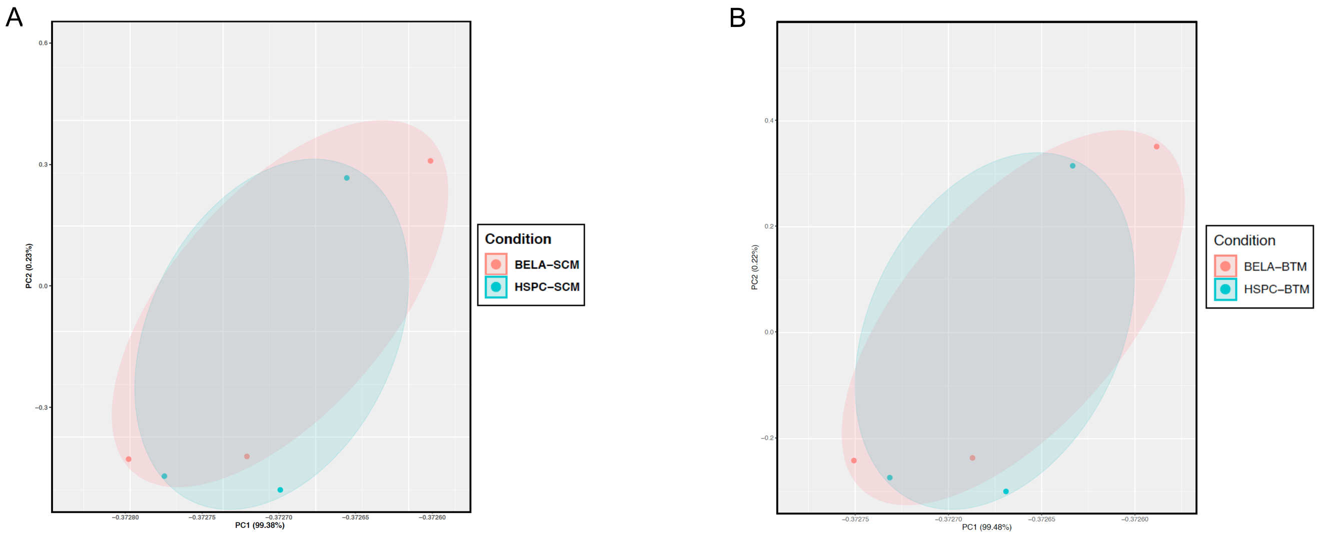

Supplementary Fig.10: Proteome profile of BEL-A cells. Principal component analysis of **(A)** BEL-A SCM with SCM HSPCs and **(B)** BEL-A BTM with BTM HSPCs.

Supplementary Fig.11

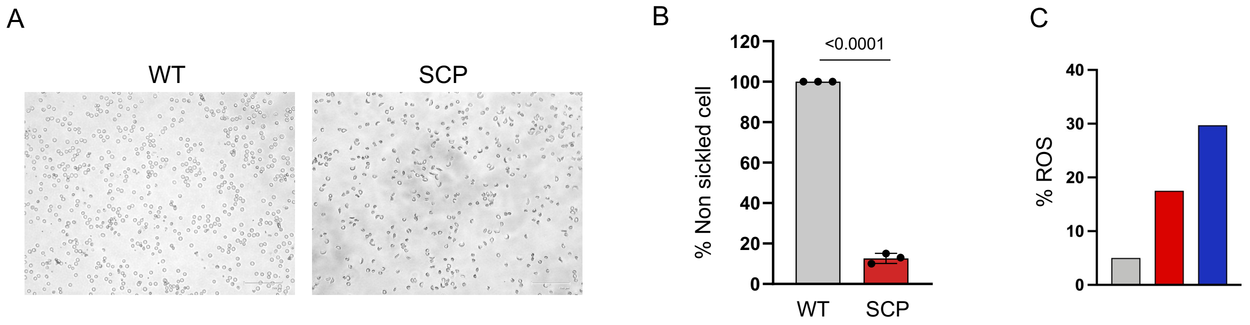

Supplementary Fig.11: (A) Representative microscopic images of sickling assay done using blood drawn from healthy individual blood (WT) and Sickle cell disease patient (SCP) and **(B)** its quantification (Mean ± S.D.). Experiments were done in three independent replicates, n=3. and statistical significance was determined by two-tailed Student's t-test. Source data is provided in source file. **(C)** Flow cytometric data for ROS levels in direct blood drawn from healthy individual (WT), sickle cell patient (SCP) and beta thalassemia patient (BTP), n=1.

Supplementary Fig.12

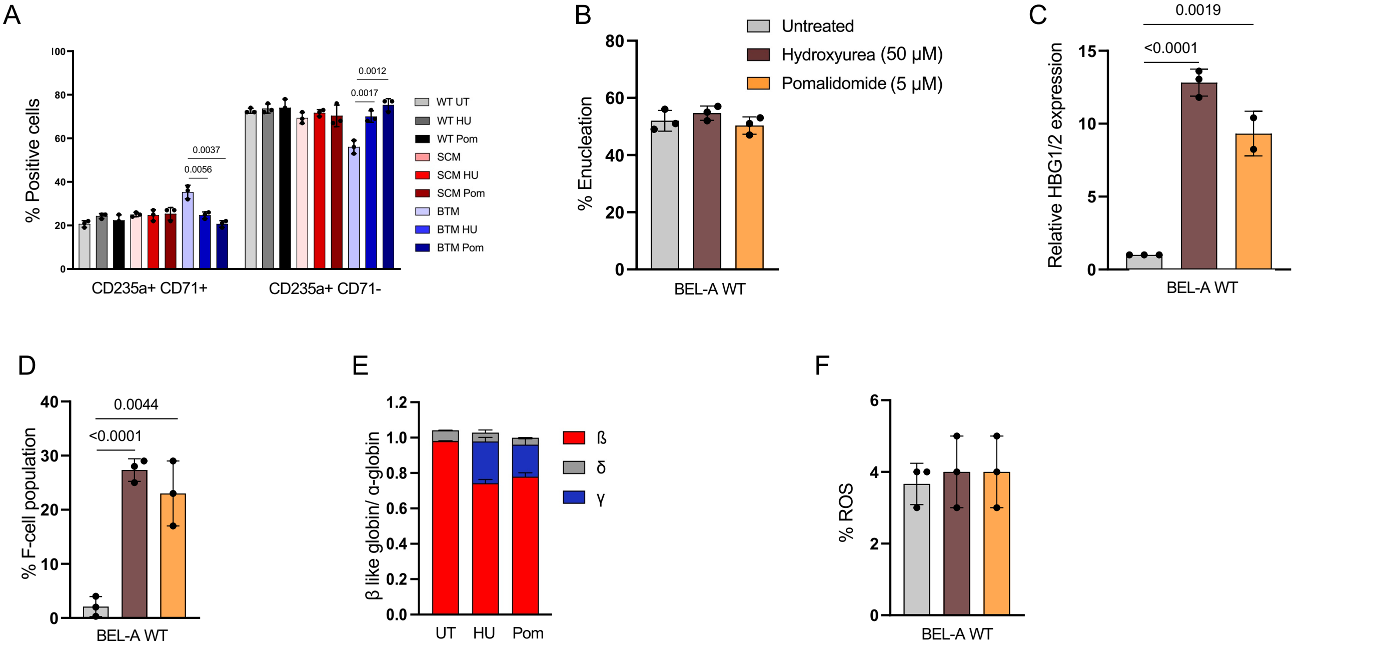

Supplementary Fig.12: Effect of Hydroxyurea and Pomalidomide on BEL-A WT cells. (A) Flow cytometric analysis of erythroid differentiation marker, CD235a and CD71 in untreated and drug treated BEL-A WT, BEL-A SCM and BEL-A BTM cells **(B)** Percent enucleation in BEL-A WT untreated and drug treated cells. **(C)** Relative HBG1/2 mRNA level done using qPCR presented as γ/α chain . **(D)** Flow cytometric analysis of F-cell population in untreated and drug treated BEL-A WT cells. **(E)** RP-HPLC data presented as total β-like goblins (β+ γ+ δ) by α-globin in untreated (UT) and hydroxyurea (HU) and Pomalidomide (Pom.) treated BEL-A WT cells. (F) Flow cytometric analysis of ROS levels in untreated and drug treated BEL-A WT cells. All experiments were done in three independent replicates and data is presented as Mean ± S.D. . Statistical significance was determined by two-tailed Student's t-test. Source data is provided in source file.

Supplementary Fig.13

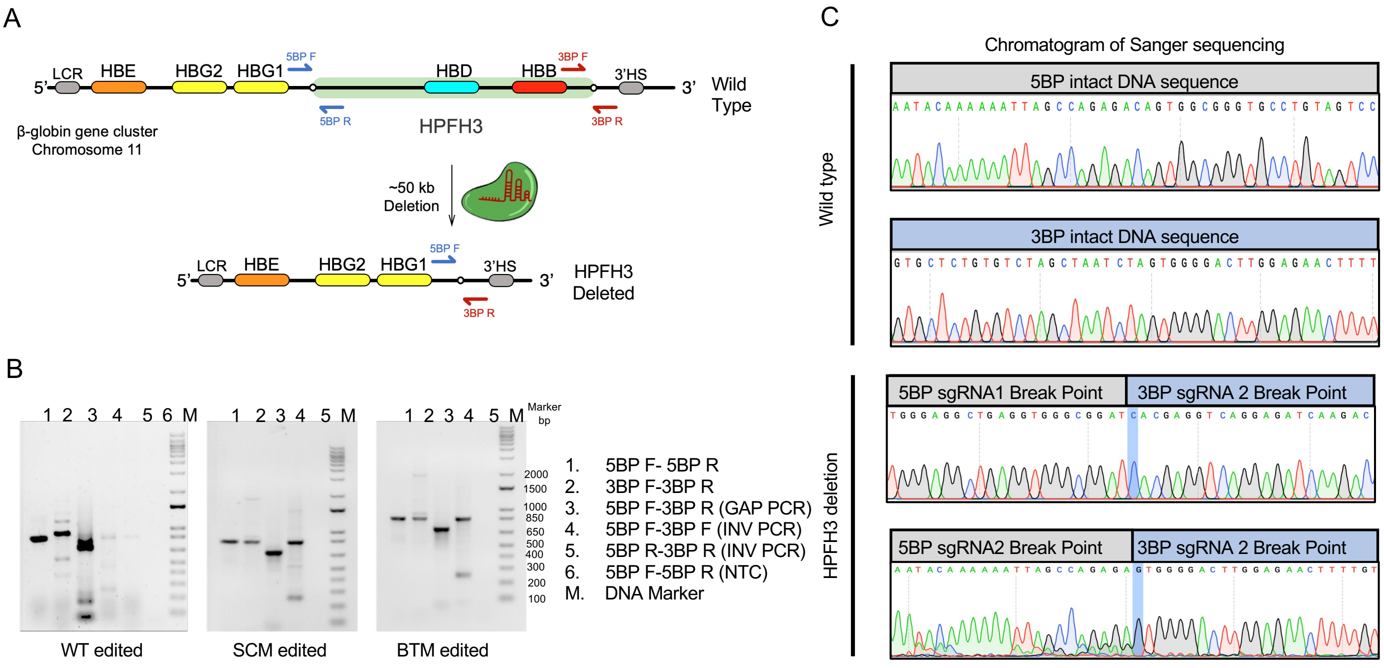

Supplementary Fig.13:Validation of HPFH3 genotype (A) Schematics depicting generation of HPFH3 genotype. (B) Agarose gel showing molecular confirmation of HPFH3 genotype in BEL-A WT, BEL-A SCM and BEL-A BTM cells. (C) Chromatograms of HPFH3 deletion confirmation through Sanger sequencing

Supplementary Fig.14

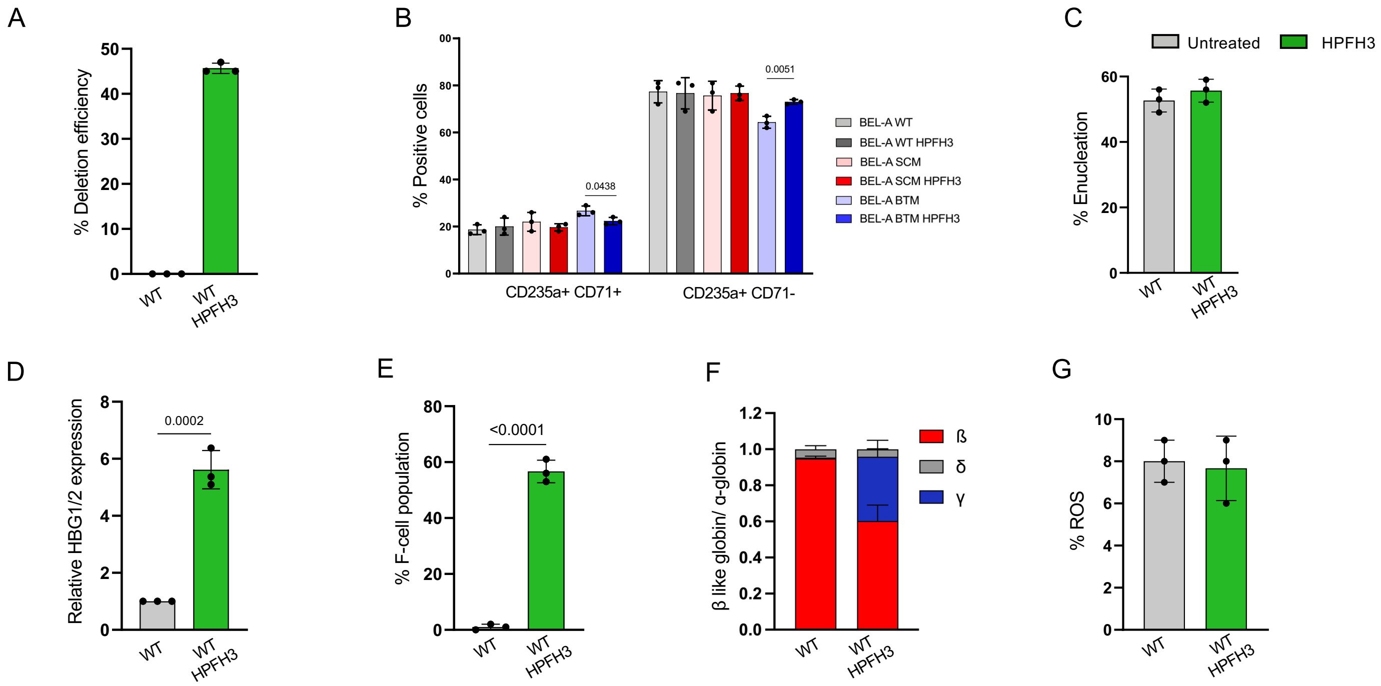

Supplementary Fig.14: Effect of HPFH3 genotype on BEL-A WT cells. (A) Deletion efficiency of HPFH3 genotype in BEL-A WT cells using ddPCR (B) Flow cytometric analysis of erythroid differentiation marker, CD235a and CD71 in unedited and HPFH3 genotype edited BEL-A WT, BEL-A SCM and BEL-A BTM cells**(C)** Percentage enucleation in BEL-A WT unedited and HPFH3 edited BEL-A WT cells. **(D)** Relative HBG1/2 mRNA level in unedited and edited BEL-A WT cells done using qPCR presented as γ/α chain . **(E)** Flow cytometric analysis of F cell population in unedited and edited BEL-A WT cells. **(F)** RP-HPLC data presented as γ globin by total β-like goblins (β+ γ+ δ) in unedited and edited BEL-A WT cells.**(G)** Flow cytometric analysis of ROS levels in unedited and edited BEL-A WT cells. All experiments were done in three independent replicates and data is presented as Mean ± S.D. Statistical significance was determined by two-tailed Student's t-test. Source data is provided in source file.

Supplementary Fig.15

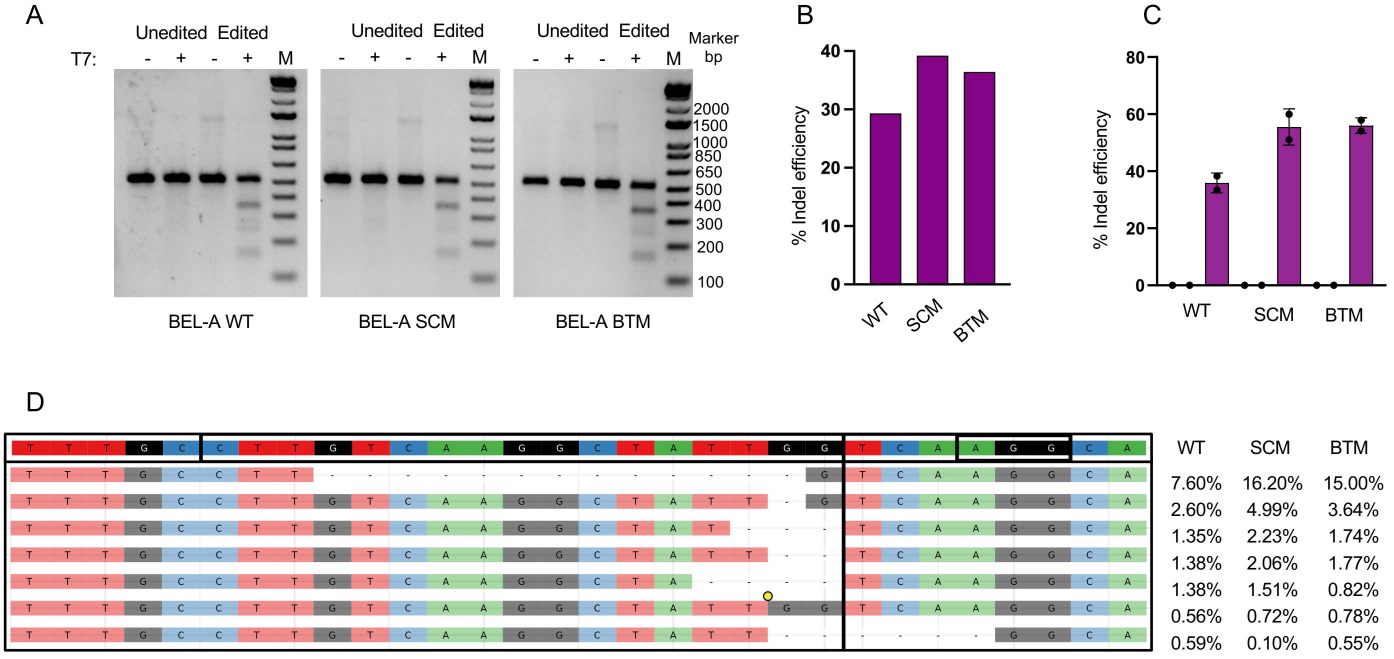

Supplementary Fig.15: Validation of HBG1/2 promoter editing (A) T7E1 assay to assess the editing efficiency of the sgRNA in BEL-A WT, BEL-A SCM and BEL-A BTM cells **(B)** TIDE analysis of editing efficiency in BEL-A WT, BEL-A SCM and BEL-A BTM, n=1 **(C)** Percentage of editing efficiency in BEL-A WT, BEL-A SCM and BEL-A BTM using Amplicon sequencing, n=2. **(D)** Frequency of Top 7 NHEJ events post editing in BEL-A WT, BEL-A SCM and BEL-A BTM cells analyzed using CrispRVariants tool.

Supplementary Fig.16

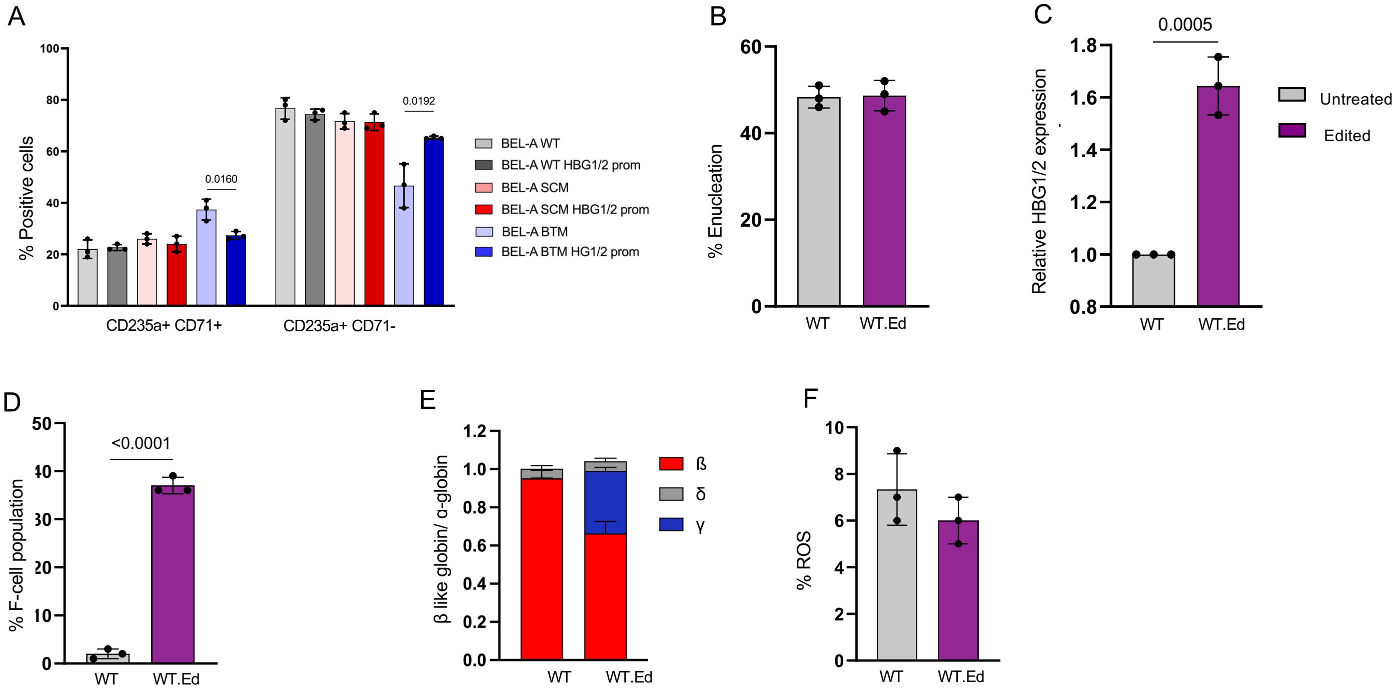

Supplementary Fig.16: Effect of HBG1/2 promoter editing on BEL-A WT cells. (A) Flow cytometric analysis of erythroid differentiation marker, CD235a and CD71 in unedited and HBG1/2 promoter edited BEL-A WT, BEL-A SCM and BEL-A BTM cells **(B)**Percentage enucleation in BEL-A WT unedited and edited cells. **(C)** Relative HBG1/2 mRNA level in unedited and edited BEL-A WT cells done using qPCR presented as γ/α chain . **(D)** Flow cytometric analysis of F-cell population in unedited and edited BEL-A WT cells. **(E)** RP-HPLC data presented as γ globin by total β-like goblins (β+ γ+ δ) in unedited and edited BEL-A WT cells. **(F)** Flow cytometric analysis of ROS levels in unedited and edited BEL-A WT cells. All experiments were done in three independent replicates and data is presented as Mean ± S.D. Statistical significance was determined by two-tailed Student's t-test. Source data is provided in source file.

Supplementary Table 1: List of sgRNA sequence used in the study

S.No.	sgRNA name	Sequence (5'-3') : 20 nt sgRNA + PAM	Reference
1	HBB sgRNA	CTTGCCCCACAGCGCAGTAA **CGG**	1
2	HBG1/2 Promoter sgRNA	CTTGTCAAGGCTATTGGTCA **AGG**	2
3	HPFH3 5BP sgRNA	CAGGCACCCGCCACTGTCTC **TGG**	In this study
4	HPFH3 3BP sgRNA	TGTGTCTAGCTAATCTAGTG **GGG**	In this study

Supplementary Table 2: Primers used for genotyping the genome editing experiments

**S.No.**	**Primer Name**	**Primer sequence (5'-3')**
1	HPFH3 3BP F	CAACAGTCCCCAGAGTGTGATGTT
2	HPFH3 3BP R	CGGCTTCACTTCTGAAGCCAG
3	HPFH3 5 BP F	GACATGGACTATTGTTCAATG
4	HPFH3 5 BP R	AAATTAAGCCTGAACAGCAAAG
5	HBG1/2 F	CCTGGACCTATGCCTAAAACA
6	HBG1/2 R	GGCGTCTGGACTAGGAG
7	HBB F(Genotyping)	GCTGTCATCACTTAGACCTCAC
8	HBB R(Genotyping)	TGAGAACTTCAGGGTGAGTCTATG

Supplementary Table 3: Sequence of Homology arms used for piggyBac constructs

**S.No.**	**Name**	**Sequence**
1	Left Homology arm (LHA)	CCGAGGTAGAGTTTTCATCCATTCTGTCCTGTAAGTAT TTTGCATATTCTGGAGACGCAGGAAGAGATCCATCTA CATATCCCAAAGCTGAATTATGGTAGACAAAACTCTTC

		CACTTTTAGTGCATCAACTTCTTATTTGTGTAATAAGAA AATTGGGAAAACGATCTTCAATATGCTTACCAAGCTGT GATTCCAAATATTACGTAAATACACTTGCAAAGGAGGA TGTTTTTAGTAGCAATTTGTACTGATGGTATGGGGCCA AGAGATATATCTTAGAGGGAGGGCTGAGGGTTTGAAG TCCAACTCCTAAGCCAGTGCCAGAAGAGCCAAGGACA GGTACGGCTGTCATCACTTAGACCTCACCCTGTGGAG CCACACCCTAGGGTTGGCCAATCTACTCCCAGGAGCA GGGAGGGCAGGAGCCAGGGCTGGGCATAAAAGTCAG GGCAGAGCCATCTATTGCTTACATTTGCTTCTGACACA ACTGTGTTCACTAGCAACCTCAAACAGACACCATGGT GCATTTAA
2	Right Homology arm (RHA-SCM)*	TTAACTCCCGTGGAAAAGTCCGCGGTTACTGCGCTGT GGGGCAAGGTGAACGTGGATGAAGTTGGTGGTGAGG CCCTGGGCAGGTTGGTATCAAGGTTACAAGACAGGTT TAAGGAGACCAATAGAAACTGGGCATGTGGAGACAGA GAAGACTCTTGGGTTTCTGATAGGCACTGACTCTCTC TGCCTATTGGTCTATTTTCCCACCCTTAGGCTGCTGGT GGTCTACCCTTGGACCCAGAGGTTCTTTGAGTCCTTT GGGGATCTGTCCACTCCTGATGCTGTTATGGGCAACC CTAAGGTGAAGGCTCATGGCAAGAAAGTGCTCGGTG CCTTTAGTGATGGCCTGGCTCACCTGGACAACCTCAA GGGCACCTTTGCCACACTGAGTGAGCTGCACTGTGAC AAGCTGCACGTGGATCCTGAGAACTTCAGGGTGAGTC TATGGGACGCTTGATGTTTTCTTTCCCCTTCTTTTCTAT GGTTAAGTTCATGTCATAGGAAGGGGATAAGTAACAG GGTACAGTTTAGAATGGGAAACAGACGAATGATTGC
3	Right Homology arm (RHA-BTM)**	TTAACTCCCGAGGAAAAGTCCGCGGTTACTGCGCTGT GGGGCAAGGTGAACGTGGATGAAGTTGGTGGTGAGG CCCTGGGCAGGTTGCTATCAAGGTTACAAGACAGGTT TAAGGAGACCAATAGAAACTGGGCATGTGGAGACAGA GAAGACTCTTGGGTTTCTGATAGGCACTGACTCTCTC TGCCTATTGGTCTATTTTCCCACCCTTAGGCTGCTGGT GGTCTACCCTTGGACCCAGAGGTTCTTTGAGTCCTTT GGGGATCTGTCCACTCCTGATGCTGTTATGGGCAACC CTAAGGTGAAGGCTCATGGCAAGAAAGTGCTCGGTG CCTTTAGTGATGGCCTGGCTCACCTGGACAACCTCAA GGGCACCTTTGCCACACTGAGTGAGCTGCACTGTGAC AAGCTGCACGTGGATCCTGAGAACTTCAGGGTGAGTC TATGGGACGCTTGATGTTTTCTTTCCCCTTCTTTTCTAT GGTTAAGTTCATGTCATAGGAAGGGGATAAGTAACAG GGTACAGTTTAGAATGGGAAACAGACGAATGATTGC

*CTG > TTA (for generating Transposase site; p.Leu>Leu), GAG > GTG (sickle cell mutation; p.Glu>Val), GCC > GCG (disruption of PAM site; p.Ala>Ala), GCC > GCG (for generating Hha1 enzyme digestion site; p.Ala>Ala)
** CTG > TTA (for generating Transposase site; p.Leu>Leu); GCC > GCG (disruption of PAM site; p.Ala>Ala), GCC > GCG (for generating Hha1 enzyme digestion site; p.Ala>Ala), G>C (beta thalassemia IVS 1-5 mutation)

Supplementary Table 4: List of Primers used for PiggyBac cloning and validation

S.No.	Primer Name	Primer sequence (5'-3')
1	LHA-F	CCTGCAGCCCAAGCTTGGATCCCCTAGGTTAACCGAGG TAGAGTTTTCATCCA
2	LHA-R	CAATTTTACGCAGACTATCTTTCTAGGGTTAAATGCACC ATGGTGTCTGT
3	RHA (SCM)-F	TACGTCACAATATGATTATCTTTCTAGGGTTAACTCCTG TGGAGAAGTCTGCGGTTACTGCGCTGTGGGGC
4	RHA (BTM)-R	TACGTCACAATATGATTATCTTTCTAGGGTTAACTCCTG AGGAGAAGTCTGCGGTTACTGCGCTGTGGGGC
5	RHA-R	TTAAGCGGCCGCATACGCGTATACTAGGTTGCAATCAT TCGTCTGTTTCCCA
6	HBB F (LHA seq)	GCTTTTTGTTCCCCCAGACAC
7	pDONOR Reverse (LHA seq)	CATTGACAAGCACGCCTCAC
8	pDONOR Forward (RHA seq)	TATGGAGATCCCTCGACCTG
9	HBB R (RHA seq)	CCCTGATTTGGTCAATATGTGT

Supplementary Table 5: Primers used ddPCR

S.No.	Primer Name	Primer sequence (5'-3')
1	HPFH3 5 BP F (ddPCR)	GCTGAGGTGGGCGGATCACG
2	HPFH3 5 BP R (ddPCR)	GGTTCACGCCATTCTCCTGC
3	HPFH3 3 BP F (ddPCR	ATCAGCAATCTGTGTCTAAC
4	HPFH3 3 BP R (ddPCR)	TGAGCTAGACACAGAGTGCT

Supplementary Table 6: List of Antibodies/dyes used in the study

S.No.	Product Name	Company	Catalogue number	Application
1	Anti-CD235 FITC	Stemcell Technologies	60152F	Flow cytometry
2	Anti-CD71 PE	Stemcell Technologies	60106PE	Flow cytometry
3	Anti-Fetal Hemoglobin Monoclonal antibody (HBF-1)	Thermofisher Scientific	MHFH05	Flow cytometry
4	Anti-Hemoglobin β antibody FITC	Santa Cruz Biotechnology	21757 FITC	Flow cytometry
5	Anti-hemoglobin α PE	Santa Cruz Biotechnology	514378 PE	Flow cytometry
6	Hoechst 33342	Invitrogen	R37165	Enucleation
7	CM-H2DCFDA (ROS)	Invitrogen	C6827	ROS
6	APC anti-human CD34 Antibody Clone 581	Biolegend	343509	B351596
7	FITC anti-human CD45 Antibody Clone H130	Biolegend	304006	B354431

Supplementary Table 7: List of primers used for qPCR

**S.No.**	**Primer Name**	**Forward Sequence (5'-3')**	**Reverse Sequence (5'-3')**	**Amplicon size**
1	HBA	CGGTCAACTTCAAG CTCCTAA	GCTCACAGAAGCCAGG AACTTG	116 bp
2	HBB	GCTCACCTGGACAA CCTCAA	CGTTGCCCAGGAGCCT GAA	100 bp
3	HBD	GCTCATGGCAAGAA GGTGCTAG	ACACCAGCACATTGCC CAAGAG	151 bp
4	HBG	CCTGTCCTCTGCCT CTGCC	GGATTGCCAAAACGGT CAC	209 bp

5	GAPDH	GAAGGCTGGGGCTC ATTT	CAGGAGGCATTGCTGA TG	138 bp
6	LMO2	GCGCCTCTACTACA AACTGGGC	CTCATAGGCACGAATC CGCTTG	109 bp
7	ZBTB7A	AAGCCCTACGAGTG CAACATCT	CAGGTCGTAGTTGTGG GCAAA	138 bp
8	GATA1	TTGTCAGTAAACGG GCAGGTA	CTTGCGGTTTCGAGTC TGAAT	179 bp
9	KLF3	ACCCAGTTCCTGTC AAGCAA	TCAGGCAATGGTGTGG AGTA	118 bp
10	EKLF	TTGCGGCAAGAGCT ACACCAAG	GTAGTGGCGGGTCAG CTCGTC	136 bp
11	SOX6	TACCTCTACCTCAC CACATAAGC	ACATCGGCAAGACTCC CTTTG	108 bp
12	FOG 1	AAGGACAGGAACCA GAACCCAG	CTCTGCTGGCTCCTTC TTCA	416 bp
13	PABPC1	AGCTGTTCCCAACC CTGTAATC	GGATAGTATGCAGCAC GGTTCTG	102 bp
14	BCL11A	CGAGCACAAACGGA AACAATG	GATTAGAGCTCCATGT GCAGAACG	253 bp

References

1. Dever, D. P. *et al.* CRISPR/Cas9 β-globin gene targeting in human haematopoietic stem cells. *Nature* **539**, 384–389 (2016).
2. Traxler, E. A. *et al.* A genome-editing strategy to treat β-hemoglobinopathies that recapitulates a mutation associated with a benign genetic condition. *Nat. Med.* **22**, 987–990 (2016).
