# Supplementary material for: Development of pathophysiologically relevant models of sickle cell disease and β-thalassemia for therapeutic studies
Source: Nat Commun. 2024 Feb 27;15:1794. doi: 10.1038/s41467-024-46036-x (PMC10899644; doi:10.1038/s41467-024-46036-x)
Supplement: Supplementary file 3 — Description of Additional Supplementary Files [file 41467_2024_46036_MOESM3_ESM.pdf]

<u>Description of Additional Supplementary Files</u>

Supplementary Data 1:

Comparison of protein levels between BEL-A SCM and BEL-A BTM with their respective HSPC counterparts. Proteomics was done

on whole cells lysates by labelling with Tandem Mass Tag (TMT) and analyzed by LC-MS/MS.

Supplementary Data 2:

Proteins levels that differed in comparison of a) BEL-A SCM versus SCM HSPCs and (b) BEL-A BTM versus BTM HSPCs.
