# Supplementary material for: Development of pathophysiologically relevant models of sickle cell disease and β-thalassemia for therapeutic studies
Source: Nat Commun. 2024 Feb 27;15:1794. doi: 10.1038/s41467-024-46036-x (PMC10899644; doi:10.1038/s41467-024-46036-x)
Supplement: Supplementary file 6 — Reporting Summary [file 41467_2024_46036_MOESM6_ESM.pdf]

nature portfolio

Corresponding author(s): Shailja Singh and Sivaprakash Ramalingam

Last updated by author(s): 31.01.2024

Reporting Summary

Nature Portfolio wishes to improve the reproducibility of the work that we publish. This form provides structure for consistency and transparency in reporting. For further information on Nature Portfolio policies, see our Editorial Policies and the Editorial Policy Checklist.

Statistics

For all statistical analyses, confirm that the following items are present in the figure legend, table legend, main text, or Methods section.

n/a	Confirmed	
☐	☒	The exact sample size (*n*) for each experimental group/condition, given as a discrete number and unit of measurement
☐	☒	A statement on whether measurements were taken from distinct samples or whether the same sample was measured repeatedly
☐	☒	The statistical test(s) used AND whether they are one- or two-sided<br>*Only common tests should be described solely by name; describe more complex techniques in the Methods section.*
☒	☐	A description of all covariates tested
☐	☒	A description of any assumptions or corrections, such as tests of normality and adjustment for multiple comparisons
☐	☒	A full description of the statistical parameters including central tendency (e.g. means) or other basic estimates (e.g. regression coefficient) AND variation (e.g. standard deviation) or associated estimates of uncertainty (e.g. confidence intervals)
☐	☒	For null hypothesis testing, the test statistic (e.g. *F*, *t*, *r*) with confidence intervals, effect sizes, degrees of freedom and *P* value noted<br>*Give P values as exact values whenever suitable.*
☒	☐	For Bayesian analysis, information on the choice of priors and Markov chain Monte Carlo settings
☒	☐	For hierarchical and complex designs, identification of the appropriate level for tests and full reporting of outcomes
☒	☐	Estimates of effect sizes (e.g. Cohen's *d*, Pearson's *r*), indicating how they were calculated

Our web collection on statistics for biologists contains articles on many of the points above.

Software and code

Policy information about availability of computer code

Data collection	For proteomic analyses, all spectra were acquired using an Orbitrap Fusion Tribrid mass spectrometer controlled by Xcalibur 2.1 software (Thermo Scientific). For flow cytomteric experminents, BD Acccuri C6 software was used. FACS Bulk and single cell sorting was done in BD Aria III. NGS data were collected using Illumina sequencing platform. Real time PCR was done using CFX384 Real Time system (BioRad)
Data analysis	Flow cytometric data was analysed using FlowJo version 10. Sanger Sequencing data was analysed using TIDE (Brinkman et al., Nucleic Acids Res. 2014 Dec 16;42(22):e1681 and NGS sequencing data was alaysed using CrispRVariant tool (Lindsay et al, Nat Biotechnol. 2016 Jul 12;34(7):701-2). All the raw files of proteomics data were searched against database Homo sapiens v110 and the contaminant database in Proteome Discoverer (PD) (version 2.2, Thermo Scientific) software using Sequest HT and MASCOT search engine. Statistical analysis was performed on GraphPad Prism version 9.5.0.

For manuscripts utilizing custom algorithms or software that are central to the research but not yet described in published literature, software must be made available to editors and reviewers. We strongly encourage code deposition in a community repository (e.g. GitHub). See the Nature Portfolio guidelines for submitting code & software for further information.

Data

Policy information about availability of data

All manuscripts must include a data availability statement. This statement should provide the following information, where applicable:

- Accession codes, unique identifiers, or web links for publicly available datasets
- A description of any restrictions on data availability
- For clinical datasets or third party data, please ensure that the statement adheres to our policy

The proteomic data generated in this study are deposited in the PRIDE database under accession code PXD044642 and processed data uploaded in Supplementary Table 1 and 2. Amplicon sequencing data generated in this study are deposited in NCBI Sequencing Reads Archive (SRA) BioProject under the accession code PRJNA1070375 (https://www.ncbi.nlm.nih.gov/sra/PRJNA1070375). Raw data for each graph presented in each figure is provided in the source data file.

Research involving human participants, their data, or biological material

Policy information about studies with human participants or human data. See also policy information about sex, gender (identity/presentation), and sexual orientation and race, ethnicity and racism.

Reporting on sex and gender	This paper excludes research studies involving human participants, it solely focuses on samples acquired from human participants. Sickle cell disease (SCD) and beta-thalassemia (BT) patients were adults, however, additional details are unavailable as the samples were completely de-identified prior to laboratory experiments. Both SCD and BT are not associated with sex-linked inheritance. Hence, sex and gender information could not be provided and also not applicable for the study.
Reporting on race, ethnicity, or other socially relevant groupings	Sickle cell disease (SCD) and beta-thalassemia (BT) patients were adults, however, additional details are unavailable as the samples were completely de-identified prior to laboratory experiments. Hence, it is not possible to provide information on race, ethnicity, or other socially relevant grouping.
Population characteristics	This paper excludes research studies involving human participants, it solely focuses on samples acquired from human participants. Sickle cell disease (SCD) and beta-thalassemia (BT) patients were adults, however, additional details are unavailable as the samples were completely de-identified prior to laboratory experiments. Both SCD and BT are not associated with sex-linked inheritance. SCD is commonly found in populations from African countries, Southeast Asia, and the Middle East. BT is most commonly found in populations from Asia, the Mediterranean basin and the Middle East.
Recruitment	Adult healthy donor volunteers were recruited anonymously. SCD and BT patients were recruited based on a clinician's diagnosis with genotype confirmation. There is an absence of potential self-selection bias and other biases. Written informed consent was obtained from donors to participate in this study. No compensation was provided to the donors.
Ethics oversight	The study was approved by the Institutional Human Ethics Committees at Institute of Genomics and Integrative Biology and Thalassemia and sickle cell society (CSIR-IGIB/IHEC/2017-18/12; TSCS-1112018; 2020-001-EMP-28) and used according to the Declaration of Helsinki. No compensation was provided to the donors. Written informed consent was obtained from donors to participate in this study.

Note that full information on the approval of the study protocol must also be provided in the manuscript.

Field-specific reporting

Please select the one below that is the best fit for your research. If you are not sure, read the appropriate sections before making your selection.

☒ Life sciences ☐ Behavioural & social sciences ☐ Ecological, evolutionary & environmental sciences

For a reference copy of the document with all sections, see nature.com/documents/nr-reporting-summary-flat.pdf

Life sciences study design

All studies must disclose on these points even when the disclosure is negative.

Sample size	No sample size was calculated. Statistical analysis for most experiments were done based on data obtained from biological triplicate, which is community default criteria.
Data exclusions	Samples were excluded only when there antibodies or reagent evidently did not worked during the experiment. In such scenarios, independent replication of the experiment was conducted unless mentioned otherwise
Replication	Each experiments were replicated two or three times as indicated in the figure legends and manuscript.
Randomization	No randomization as the final chosen clonal population was based on confirmed Sanger sequencing and the phenotypes.
Blinding	Not blinded as same gating was used for analysis. Two independent investigators conducted the experiments for all groups. Covariation is not applicable for the study

Reporting for specific materials, systems and methods

We require information from authors about some types of materials, experimental systems and methods used in many studies. Here, indicate whether each material, system or method listed is relevant to your study. If you are not sure if a list item applies to your research, read the appropriate section before selecting a response.

Materials & experimental systems

n/a	Involved in the study
☐	☒ Antibodies
☐	☒ Eukaryotic cell lines
☒	☐ Palaeontology and archaeology
☒	☐ Animals and other organisms
☒	☐ Clinical data
☒	☐ Dual use research of concern
☒	☐ Plants

Methods

n/a	Involved in the study
☒	☐ ChIP-seq
☐	☒ Flow cytometry
☒	☐ MRI-based neuroimaging

Antibodies

Antibodies used

1) Anti-CD235 FITC, Clone 2B7 (Cat #60152FI ; Lot #100046498; Stemcell Technologies)
2) Anti -Human CD71 (Transferrin Receptor) Antibody, Clone OKT9 (Cat # 60106PE; Lot #1000072401; Stemcell Technologies)
3)Anti-Fetal Hemoglobin monoclonal antibody (HBF-1) (Cat # MHFH05; Lot # 2365782; Thermofisher Scientific)
4) Anti-hemoglobin (37-8) Antibody FITC (Cat #sc-21757 FITC ; Lot #H0519 ; Santa Cruz Biotechnology)
5)Anti- hemoglobin (D-4) PE (Cat #sc-514378 PE ; Lot #H3019 ;Santa Cruz Biotechnology)
6)APC anti-human CD34 Antibody Clone 581 (Cat # 343509; Lot #6351596 ; Biolegend)
7) FITC anti-human CD45 Antibody Clone H130 (Cat #304006 ; Lot #6354431 ; Biolegend)
8) Hoechst 33342 (Cat #R37165 ; Lot #2139328; Invitrogen)
9) CM-H2DCFDA (ROS) (Cat #C6827 ; Lot #2400831 ; Invitrogen)

Validation

Validation of each antibody is provided on the manufacturers website.

Eukaryotic cell lines

Policy information about cell lines and Sex and Gender in Research

Cell line source(s)

The BEL-A cell line was made at University of Briston in Prof. Jayne Fraynes lab from adult bone marrow CD34+ cells. The generation and the charcterization of the line shown in Trakarnsanga et al, Nat Commun. 2017 Mar 14;8:14750

Authentication

Authentication of the line is mentioned in Trakarnsanga et al, Nat Commun. 2017 Mar 14;8:14750 at morphological, molecular and proteomic levels.

Mycoplasma contamination

All cell lines were tested negative for Mycoplasma

Commonly misidentified lines
(See ICLAC register)

No commonly misidentified lines were used in the study

Plants

Seed stocks

Report on the source of all seed stocks or other plant material used. If applicable, state the seed stock centre and catalogue number. If plant specimens were collected from the field, describe the collection location, date and sampling procedures.

Novel plant genotypes

Describe the methods by which all novel plant genotypes were produced. This includes those generated by transgenic approaches, gene editing, chemical/radiation-based mutagenesis and hybridization. For transgenic lines, describe the transformation method, the number of independent lines analyzed and the generation upon which experiments were performed. For gene-edited lines, describe the editor used, the endogenous sequence targeted for editing, the targeting guide RNA sequence (if applicable) and how the editor was applied.

Authentication

Describe any authentication procedures for each seed stock used or novel genotype generated. Describe any experiments used to assess the effect of a mutation and, where applicable, how potential secondary effects (e.g. second site T-DNA insertions, mosiacism, off-target gene editing) were examined.

Flow Cytometry

Plots

Confirm that:

☒ The axis labels state the marker and fluorochrome used (e.g. CD4-FITC).

☒ The axis scales are clearly visible. Include numbers along axes only for bottom left plot of group (a 'group' is an analysis of identical markers).

☐ All plots are contour plots with outliers or pseudocolor plots.

☒ A numerical value for number of cells or percentage (with statistics) is provided.

Methodology

Sample preparation	For flow cytometry based analysis experiments:<br>Surface Markers/ dye: 1-3 x105 cells were harvested on day 12 for BEL-A and Day 21 for HSPCs of erythroid differentiation and were stained for anti-CD71-PE, Glycophorin-A-FITC (GYPA), Hoechst 33342, CM-H2DCFDA as described in the manuscript.<br>For intracellular staining: 1-3x105 erythroid differentiated cells were fixed with 4% formaldehyde (Sigma Aldrich) for 10 minutes, permeabilized with 0.1% Triton X-100 (Sigma Aldrich) for 5 minutes. Cells were then washed with PBS supplemented with 2% FBS and stained with anti-HBF-1 (Thermo Fisher Scientific), anti-hemoglobin antibody (Santa Cruz Biotechnology) and Anti-hemoglobin antibody (Santa Cruz biotechnology) as described in the manuscript.<br>For cell sorting experiments:<br>3X10^6 cells were taken and PBS washed. Population of interest was then sorted as described in the manuscript
Instrument	BD Accuri C6 Flow cytometer and BD Aria III.
Software	For FACS BD Aria III: BD FACSDiva<br>For analysis from BD Accuri C6: BD Accuri C6 software and FlowJo version 10
Cell population abundance	For the generation of the BEL-A SCM and BEL-A BTM cell lines, eGFP+dTomato+BFP- (Double Positive) cells were sorted after Neon electroporation of Donor and sgRNA plasmids. Post Transposase treatment, eGFP-dTomato- (double negative) cells were sorted.
Gating strategy	The cell sorting was done in a hierarchical manner, firstly, FSC-A/SSC-A gating was applied to exclude cell debris and acquire target population of cells followed by gating of cells in FSC-W/FSC-H and SSC-W/SS-H to exclude doublets. For the generation of the BEL-A SCM and BEL-A BTM cell lines, eGFP+dTomato+BFP- (Double Positive) cells were sorted after Neon electroporation of Donor and sgRNA plasmids. Subsequently, BFP- cells were gated and amongst them eGFP+ and dTomato+ cells were selected and sorted in BD FACSAriaIII. eGFP+dTomato+BFP- cells were allowed to amplify and were single cell sorted and expanded. Similarly, post Transposase treatment, FSC-A/SSC-A gating was applied to exclude cell debris and acquire target population of cells followed by gating of cells in FSC-W/FSC-H and SSC-W/SS-H to exclude doublets which was followed by gating eGFP-dTomato- (double negative) population and were sorted at single cell in 96 well plate

☐ Tick this box to confirm that a figure exemplifying the gating strategy is provided in the Supplementary Information.
